# A framework for assessing health system resilience in an economic crisis: Ireland as a test case

**DOI:** 10.1186/1472-6963-13-450

**Published:** 2013-10-30

**Authors:** Steve Thomas, Conor Keegan, Sarah Barry, Richard Layte, Matt Jowett, Charles Normand

**Affiliations:** 1 Centre for Health Policy and Management, Trinity College Dublin, 3-4 Foster Place, College Green, Dublin 2, Ireland; 2 Economic and Social Research Institute, Whitaker Square, Sir John Rogerson’s Quay, Dublin, Ireland; 3 WHO Barcelona Office for Health Systems Strengthening, Marc Aureli 22-36, E-08006, Barcelona, Spain

**Keywords:** Resilience, Health systems, Sustainability, Economic crisis, Ireland

## Abstract

**Background:**

The financial crisis that hit the global economy in 2007 was unprecedented in the post war era. In general the crisis has created a difficult environment for health systems globally. The purpose of this paper is to develop a framework for assessing the resilience of health systems in terms of how they have adjusted to economic crisis. Resilience can be understood as the capacity of a system to absorb change but continue to retain essentially the same identity and function. The Irish health system is used as a case study to assess the usefulness of this framework.

**Methods:**

The authors identify three forms of resilience: financial, adaptive and transformatory. Indicators of performance are presented to allow for testing of the framework and measurement of system performance. Both quantitative and qualitative methods were used to yield data for the Irish case study. Quantitative data were collected from government documents and sources to understand the depth of the recession and the different dimensions of the response. Semi-structured interviews were conducted with key decision makers to understand the reasons for decisions made.

**Results:**

In the Irish case there is mixed evidence on resilience. Health funding was initially protected but was then followed by deep cuts as the crisis deepened. There is strong evidence for adaptive resilience, with the health system showing efficiency gains from the recession. Nevertheless, easy efficiencies have been made and continued austerity will mean cuts in entitlements and services. The prospects for building and maintaining transformatory resilience are unsure. While the direction of reform is clear, and has been preserved to date, it is not certain whether it will remain manageable given continued austerity, some loss of sovereignty and capacity limitations.

**Conclusions:**

The three aspects of resilience proved a useful categorisation of performance measurement though there is overlap between them. Transformatory resilience may be more difficult to assess precisely. It would be useful to test out the framework against other country experiences and refine the measures and indicators. Further research on both the comparative resilience of different health systems and building resilience in preparation for crises is encouraged.

## Background

### Context and scope

The financial crisis that hit the global economy in 2007 was unprecedented in the post war era. It quickly transformed into an economic crisis which saw European Union real Gross Domestic Product (GDP) shrink by 4.3 per cent in 2009, the sharpest contraction in its history [1]^a^. Some countries are experiencing persistent extreme austerity and have needed bailouts from international financial authorities to manage their spiralling deficits and debt [2]. In general the crisis has created a difficult environment for health systems globally as available resources are constrained or shrink and services are overburdened often leaving the vulnerable at particular risk [3]. Austerity easily leads to fewer services, poorer access and more financial burden on households at exactly the wrong time.

The purpose of this paper is to develop a framework for assessing the resilience of health systems in terms of how they have adjusted to economic crisis. The focus of the paper is health system performance under austerity rather than on how the health system impacts the economy. The latter is important as the health system is a major employer particularly in rural areas, but this concern lies largely outside the scope of the article. Key questions relate to how well the health system has continued to function in the face of austerity and how well the vulnerable have been protected. It has particular relevance for the current European context or where scarcity is pronounced and economic sovereignty threatened. The term resilience has been drawn from the study of socio-ecological systems where fragility, survival and appropriate management of critical situations are a key topic of research [4]. Resilience can be understood as the capacity of a system to absorb change but continue to retain essentially the same identity and function [4].

Ireland will be used as a case study to assess the usefulness of this framework and what it can show us about health system performance in time for crisis. As a small open economy, Ireland was particularly exposed to and affected by the global economic and financial crisis. Further, domestic mismanagement of the Irish economy worsened the situation. Years of access to cheap credit and minimum government oversight in Ireland saw the development of an unsustainable property bubble. This contributed to an internal banking collapse. The bank guarantee scheme announced in September 2008 coupled banking and sovereign debt in Ireland and placed massive strain on the State’s finances. Further, taxation policy which had focussed on indirect taxes proved disastrous for government revenues in the recession [5]. In late 2010 the government was forced to accept an EU/IMF/ECB bailout totalling €85 billion [6]. The NESC (National Economic and Social Council) in 2009 has described Ireland as undergoing a five-fold crisis, i.e. a combination of a banking crisis, public finance crisis, an economic crisis, a social crisis and a 'reputational’ crisis [7]. The severe nature of Ireland’s crisis provides a useful test for the resilience approach.

### Health policy in a time of crisis

Parry and Humphries and Stuckler et al. emphasise the importance of government intervention to mitigate the impact of economic contraction [3,8]. More specifically, Musgrove argues that a good health policy, or change in existing health policy, would maintain (or even extend) services most essential to health due to the 'fluctuation of need’ from the private sector to the public sector [9]. More generally, healthcare spending should be counter-cyclical to cope with the substitution of private for public healthcare services in times of crises [9]. The World Bank echoes these sentiments arguing that 'the fundamental objective of health policy during a crisis is to maintain/improve access to essential services by the population, and especially the poor and vulnerable’ [10]. In addition preserving health sector spending and employment may provide economic benefits.

However, this counter-cyclical funding is rarely the case. Musgrove notes the absence of a 'counter-cyclical commitment’ when analysing the policy response of several Latin American and Caribbean countries following the 1980’s debt crisis, which were partly related to the conditionalities associated with IMF/WB loans [9]. The World Bank, examining evidence of previous financial crises in Argentina, Indonesia, Thailand and the Russian Federation, highlight the 'pro-cyclical declines’ in health spending [10]. Total, public and out-of-pocket health spending all decreased in per capita terms in all these countries, taking many years to reach pre-crisis levels.

Nevertheless, stakeholder power and expectations which tend to preserve the status quo are weakened in a recession which can give scope for radical reform of a health system and this can give scope for change [8,11].

### Resilience and evaluating system performance

There are several frameworks for assessing health system performance (such as World Health Organisation [12]; McPake & Kutzin [13]). Nevertheless, the core features or values of these tend to overlap and relate to allocative efficiency (maximising the impact of health promoting interventions across a broad range of activities), technical efficiency (optimal combination of resources in any *one* activity to produce maximum output at minimum cost), equity (fairness of financing and access, especially for the most vulnerable) and acceptability/responsiveness to stakeholders [14]. Such criteria are important to use in reviewing health system performance at any time, whether in recession or not. Nevertheless in a time of economic contraction, when past and present taxation and regulatory policies create conditions of scarcity, some additional factors assume more importance, such as sustainability.

The issue of sustainability of a system is of paramount concern, particularly when finance is scarce. Indeed, a standard measure of health system performance is financial sustainability. There are two prevailing definitions. The first discusses the financing of the health sector in relation to its dependency on *external* resources [15]. Of major concern here is the flow of foreign donor funds into the health system or the degree of debt that countries are accruing to finance health. The second definition is concerned with the sufficiency, predictability and regularity of sources of finances in the health sector [13]. Such an interpretation of financial sustainability is less concerned with the source of funds for financing a health sector, and more interested in a steady future flow of finances. These definitions are a helpful starting point in determining when there are key problems with financing and also can highlight trends in sustainability. They do not however offer any insights into managing the problem and understanding the implications, causalities and dimensions of a loss of financial sustainability.

Broader approaches to health system sustainability are needed. “A health service is considered sustainable when operated by an organizational system with the long-term ability to mobilize and allocate sufficient resources for activities that meet individual or public health needs.” [16]. This definition focuses on two aspects: the ability to raise sufficient funds over the long-term and the ability to use these resources in a way that meets needs. Most definitions focus on these elements of sufficiency of resource generation and effectiveness in use [17-19]. Nevertheless, other definitions also focus on the capacity and commitment of government, as it is government which mobilises the majority of resources (or facilitates their mobilisation), develops policy and allocates resources [20-22]. Hence an appropriate analysis of sustainability needs also to focus on the governance of a health system and its ability to respond to resource shortages, alongside the capacity of the system to mobilise resources and deploy them effectively.

As noted earlier, the study of socio-ecological systems examines the concept of *resilience*. This can be defined as “the capacity of a system to absorb disturbance and reorganise while undergoing change so as to still retain essentially the same function, structure, identity and feedbacks” [4]. There are two further concepts that deal with governance of the system and echo some of the concerns outlined in the analysis of sustainability. *Adaptability* is the capacity of actors in the system to influence or manage resilience so that the system does not shift away from its core function and structure. Relatedly, *transformability* is the capacity of actors to create a fundamentally new system when conditions make the existing system untenable. There is then a key tension in government between adaptability and transformability or between: “maintaining the resilience of a desired current configuration in the face of … shocks and simultaneously building a capacity for transformability, should it be needed” [4].

The above concepts provide useful insights into the factors which affect performance and decision-making when circumstances change and the ability of a system to cope with change. However this needs to be applied more precisely to the health system and to the economic contraction. In this case resilience might be better understood as the capacity of a health system to deal with economic contraction and reorganize so as to retain essentially the same policies and functions.

Given the need to preserve funding but also to manage scarcity and to consider transformation, there may helpfully be three forms of resilience:

• **Financial resilience**: the protection of funds for health care, and particularly that of the vulnerable, in the face of economic contraction.

• **Adaptive resilience**: the ability of government and providers to manage the system with fewer resources, through efficiencies, while not sacrificing key priorities, benefits, access or entitlements.

• **Transformatory resilience**: the ability or capacity of government to design and implement desirable and realistic reform when the current organisation, structures and strategies are no longer feasible.

It is possible that there may be overlaps or tensions between these forms of resilience. For instance, some types of adaptive resilience might be close to transformation. Alternatively, focussing too much on efficiency gains might divert capacity away from transformation. Another possible dynamic could be that the three forms of resilience represent a sequence of strategic response e.g. government’s first seek to protect funding, then to make efficiencies and finally attempt to overhaul the system in the face of prolonged resource shortages.

## Methods

It is important to operationalise the three elements of resilience in terms of useful indicators to allow for testing of the framework, measurement of system performance and cross-country comparison. Useful indicators may relate to the following:

Financial resilience:

• Protection of overall levels of health funding (public and private) as the crisis develops

• Protection of health funding compared to economic decline, to overall government spending and with other spending sectors;

• Protection of service provision over administration;

• Protection of the poor, sick and old through funding of their entitlements.

Adaptive resilience:

• Reduction of Unit costs (salaries, wages, fees)

• Increase in system productivity (Average length of stay, proportion of day cases in acute care)

• Reduction in staffing with no commensurate reduction in service.

• Protection of services (no loss of entitlements or rationing by volume).

• Achievement of stated targets.

Transformatory resilience:

• Clear specification of reforms

• Evidence base for reforms

• Progress toward reforms

• Organisational Capacity to achieve/manage reform

• System capacity to implement reform

The methods used to assess the resilience of the Irish health system were both quantitative and qualitative as appropriate to the nature of the topics of inquiry. Quantitative data were collected to understand the depth of the recession and the different dimensions of the response from government and other key parties in the health system. To help this, all key government budget and policy documents published from 2008 to 2012 have been reviewed from the Health Service Executive (HSE), the Department of Finance and the Department of Health [23-30]. The HSE in particular produces monthly reports on key financial, human resource, coverage and service indicators and these have been accessed and analysed with key performance metrics drawn from here. The analysis of adaptive resilience is framed in terms of output, rather than outcome, measures partly because of data availability in the published reports and that consequent changes in outcomes are not yet evident. In addition qualitative data were collected to understand the reasons for decisions and the decision-making processes in response to the economic and financial crisis and to get insight into the questions of capacity and reform. To do this the authors conducted semi-structured interviews with key decision makers in the Department of Health, Department of Finance and the HSE to ensure different perspectives were forthcoming.

Ethical approval for the Resilience Project and related research was given by the Ethics Committee of the Centre for Health Policy and Management, School of Medicine, Trinity College Dublin.

## Results and discussion

### Applying the framework: reflections on the resilience of the Irish health system

This section applies the framework to assess specific aspects of resilience in the Irish case study. The overall timeline of the government health system response to the recession in Ireland is shown in Table 1, noting key budgetary and policy decisions from 2008 to the end of 2011. It is in essence a summary of the management of the system through the recession and so will be referred to in the discussion of the measures of each type of resilience.

**Table 1:** Budget policy timeline 2008-2011

**2008**	**2009**			**2010**	**2011**	
**Emergency budget (Oct)**	**Supplementary budget (April)**	**Supplementary budget (November)**	**Budget (Dec)**	**Budget (Dec)**	**New programme for government**	**Budget (Dec)**
(i) Without Medical Cards: Increased Charges for IP Beds: Increased ED Charges; Increased Long-Stay Charges; Increased deductibles for drug payment scheme over-70s. Overall Health Budget for 2009 up by €200 million (1% increase)	Capital spending reduced by 26% Tax relief on nursing homes and hospitals ended	Extra funds made available to cover extra medical cards needed (€230 million)	Savings of over €1 billion (€4bn from total budget):	Savings of €746 million (€2.2 billion from total budget). Cut of 6.6% to HSE:	Commitment to UHI single tier system	Savings of €543 million (€2.2 billion from total budget)
(ii) With Medical Cards: removal of entitlement for IP Beds: Increased ED Charges; Increased Long-Stay Charges; Increased deductibles for drug payment scheme over-70s. Overall Health Budget for 2009 up by €200 million (1% increase)			• Wage Reductions (515%) and lower contract fees (€659 million)	• Voluntary redundancy and early retirement (€123 million)		• Pay cost containment (reduction in staffing, overtime, agency costs etc.) €145m
			• Introduction of 50c item charge on prescriptions for medical card holders	• Cuts in drug spending and fees (€380 million)		• Reduction in procurement costs €50m
			• Cut of €30million in spending on dentistry for those on medical cards	• Cuts in non-core pay costs, reduced agency and locum staffing (€200 million)		• Increased generation and collection of private income-€143m
			• Increase drug reimbursement threshold to €120 per month	• Administration Cuts (€43 million)		• Demand led Schemes pharmaceutical reductions, DPS increase from €120-132 per month etc. (€124m)
						+ making good the hospital deficits (€200m)

Source: Government of Ireland, Budget Statistics.

### Financial resilience

**Figure 1:**
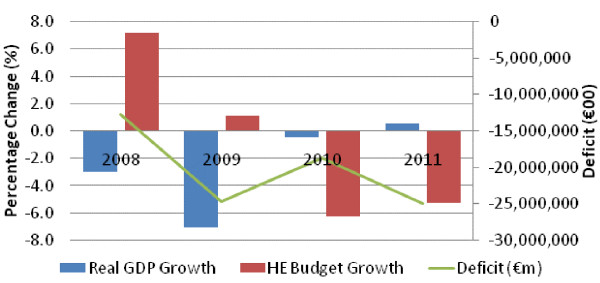
**Percentage change in GDP, the health budget, and change in the government deficit (2008–2011).** Source: EuroStat/Irish government budgetary accounts.

In the first two years of economic recession, public health expenditure appears relatively resilient when measured against changes in GDP (which declined by 3% in 2008 and 7% in 2009). There is some evidence of counter-cyclical spending in terms of extra funding for demand led-schemes such as the medical card which gives free access to the poor. Nevertheless, beyond 2009 there is little evidence of financial resilience in terms of public health spending protection with budgets being cut by over €2 billion. This is not surprising given the size of the fiscal correction required (Figure 1).

Still, relative to other departments, the Health and Children Ministerial Group’s proportion of total budgetary allocation (excluding funds spent on welfare payment through Social Protection), grew between 2008 and 2011, suggesting a form of relative protection of health expenditure. Within the Health and Children Ministerial Group there was also a shift towards protection of current expenditure and proportionately increasing the allocation to the HSE, the provider of front-line services.

Up to 2012, HSE have managed to keep their overall budget balanced, but this masked a serious problem of overruns in hospital spending as budgets fell but political pressure on waiting lists forced more hospital activity. Also there has been demand switching behaviour of households away from the private health sector toward the public system. The latter is evidenced by a fall in the numbers with supplementary private insurance (from 52% in 2006 to 46% in 2012).

Despite the strain being placed on finances, the Government has continued largely to protect access to services for the poor through the worst of the crisis. No change was made to the eligibility criteria for medical cards, resulting in a sharp increase in the number entitled to free health services by over 300,000 between 2008 and 2011 as household income levels declined.

While the poor have been reasonably protected from increases in out-of-pocket expenses over the course of the crisis, the sick and the old have been less so. This is evidenced by the fact that the deductible for the Drug Payment Scheme (DPS) has been increased three times between the Dec 2008 and Dec 2011 Budgets. This has led to an aggregated deductible increase from €90-€132 per month. While those who have a specific set of conditions are covered for free drugs under the Long Term Illness Scheme, this has not been updated since 1975 and excludes many common conditions that require significant drug costs [31]. Furthermore the removal of universal entitlement to free care in the public system for the over 70s was one of the first government responses to the crisis.

### Adaptive resilience

The data reveal that many efficiency gains were made in the recessionary period. Critical to curbing and reducing expenditure in Ireland was the advancement of the Value for Money (VfM) programme by the HSE. The VfM programme targeted efficiency savings in drug procurement, transport, contracting out, advertising and maintenance, without impacting on essential services. The VfM framework was initially designed to save €500 million between 2007 and 2010. However, savings for this period actually amounted to €687 million, significantly exceeding the original target [24].

The December 2009 Budget also reduced wages across the public sector workforce by 5-15% resulting in €659 million in health related savings. Similarly, in February 2009, emergency legislation (the Financial Emergency Measures in the Public Interest) was introduced which allowed State agencies to seek a reduction of 8% on all professional fees. This legislation allowed the HSE to announce cuts to pharmacy fees by 24-34% with effect of 1 July [32]. This was expected to save €53 million in 2009 and €133 million per annum thereafter.

Allied to the reduction in wages and fees, the Moratorium on Recruitment introduced in March 2009 focussed on reducing overall staff numbers. Under this arrangement, the HSE was expected to reduce staff by 6,000 WTEs between 2009 and 2013. At the end of 2010 WTEs stood at 107,972, a reduction of 3,798 WTEs since March 2009. The largest reduction between December 2008 and December 2010 has been in General and Support Staff (-9.58%), Nurses (-4.21%) and Management and Admin Staff (-3.71%). This amounted to an absolute cut of 1,605 WTE for nurses, the highest out of any category. While certain front-line staff grades have been exempted from this moratorium, nurses have not [24]. This may compromise performance with respect to loss of institutional memory even where services are maintained.

Analysis of key performance metrics shows overall improvements, albeit in the short run and generally below target. Measures of acute sector sustainability, such as day case ratios, day case surgery rates and average length of stay all saw improvements [24]. In terms of acute sector performance this is especially pertinent given the increase in activity above targeted levels. This evidence suggests that the health system performed well in adapting to the significant reductions in expenditure and staffing.

Interview data also supports this view with one policy maker noting that:

'The effect on services has been surprisingly small. The system is more resilient than it appears. It could also be the case that there was a lot of flab in the system’.

Nevertheless, a prevailing sentiment from the interviews is that:

“We have gone so far with efficiencies. Now we have to look at service cuts”.

### Transformatory resilience

The need for major reform was highlighted in the qualitative interviews:

“For sustainability we need new responses (how do we deliver care, what is the burden of responsibility etc.) … this is an opportunity for fundamental change”.

The Programme for Government outlines for the first time in the Irish state the principle of universal access to health care through a Universal Health Insurance system, drawing on the Dutch model [33]. The policy claims that there is much that needs to be done to reorientate the system, including moving to a contracting model for purchasing health care. The evidence base for the specific design of reform is questionable [5]. Regardless, the extent of change requires strong governance capacity. There is as yet no published detailed road-plan on how to do this implying limited capacity to do much more than cast vision. As policy makers noted:

'There isn’t going to be the capacity to deliver that wider reform within the resources in the system’.

“The key question here is, is the management of the health environment capable of delivering the changes necessary… The management of resources in the health system is in decline as senior management leave and are not replaced. The capacity of responding to the challenge for change in service delivery is one of the key limiters of getting maximum benefit from this recessionary time.”

One key informant commented:

“The New Programme for Government has an 'entirely different focus’ – Universal Primary Care and Universal Health Insurance - but Government knows we are no-longer masters of our own destiny – so how these can be delivered is a question.”

The feeling within the healthcare sector is that health system transformation has been playing second-fiddle to more immediate goals such as expenditure reduction and technical efficiency savings. There are big questions over the ability of the system to manage and implement change within this context.

### Discussion

The three aspects of resilience proved a useful categorisation though there is overlap between different elements and in some cases it may be difficult to distinguish between them e.g. elements of transformation and adaptation. While financial resilience and adaptive resilience are amenable to specific quantitative measures, transformatory resilience is broader, more qualitative and therefore more difficult to assess precisely. It would be useful to test out the framework against other country experiences and refine the measures and indicators. It would be expected that those countries that retain economic sovereignty and have room for manoeuvre with their debt levels will be more financially resilient.

Certainly, in the Irish case there seems to have been a phasing of resilience, with financial resilience initially preserved but then lost as the crisis continued. There is evidence for adaptive resilience in Ireland, with the health system showing some benefit from the recession, though further gains are uncertain and further cuts in entitlements and services are likely. The prospects for building and maintaining transformatory resilience are unsure. While the direction of reform is clear, and has been preserved to date, it is not certain whether it will remain manageable given continued austerity, capacity limitations and some loss of sovereignty to the troika (International Monetary Fund, European Union and the European Central Bank).

A key limitation of the approach in this paper is that it ignores the economic impact of public sector employment and thus, for adaptive efficiency, portrays the reduction of numbers of staff or of public sector salary levels as a good example of adaptation to fewer resources. While this may be true in terms of preserving the functioning of the health system in the short run it does not consider the economic impact of lower employment levels. These in turn will have an impact on economic activity, future taxation and the availability of funds for the health system in the future.

A further limitation may be that health systems which have more room for cuts, in terms of carrying inefficiency, may appear to have more adaptive resilience. While deliberate inefficiency is not optimal, it may be that very efficient service delivery is not flexible to changing circumstances. This raises the question of what does it take for a health system to be prepared in advance for a time of austerity and this needs additional research.

## Conclusions

This paper has developed a framework for assessing the resilience of health systems in economic crisis. In particular, the framework suggests evaluating three aspects of resilience in relation to the protection of health financing, adaptation to fewer resources through improved efficiency and transformation to a new system design. This framework was tested out against the case study of Ireland. Further research on both the comparative resilience of different health systems and building resilience in preparation for crises would be most useful.

## Endnote

^a^Some commentators question the usefulness of GDP in an Irish context as a measure of economic activity given the presence of multinationals and the repatriation of profits to non residents. Nevertheless, both GNP and GDP fell sharply over this period.

## Abbreviations

DPS: Drug payment scheme; GDP: Gross domestic product; HSE: Health service executive; UHI: Universal health insurance; WTE: Whole time equivalents; VfM: Value for money.

## Competing interests

The authors declare that they have no competing interests.

## Authors’ contributions

ST conceived the approach, led the framework development and drafted the research paper. CK led the writing and researching of quantitative data around the Irish system. SB conducted, analysed and wrote up stakeholder interviews. RL, MJ and CN inputted into the conceptual development and applicability of measures and contributed to drafts. All authors read and approved the final manuscript.

## Pre-publication history

The pre-publication history for this paper can be accessed here:

http://www.biomedcentral.com/1472-6963/13/450/prepub
